# Eye-Catching Microbes—Polyphasic Analysis of the Microbiota on Microscope Oculars Verifies Their Role as Fomites

**DOI:** 10.3390/jcm9051572

**Published:** 2020-05-22

**Authors:** Birgit Fritz, Karin Schäfer, Melanie März, Siegfried Wahl, Focke Ziemssen, Markus Egert

**Affiliations:** 1 Faculty of Medical and Life Sciences, Institute of Precision Medicine, Microbiology and Hygiene Group, Furtwangen University, Jakob-Kienzle-Strasse 17, 78054 Villingen-Schwenningen, Germany; birgit.fritz@hs-furtwangen.de (B.F.); karin.schaefer@hs-furtwangen.de (K.S.); melanie.maerz@hs-furtwangen.de (M.M.); 2 Carl Zeiss Vision International GmbH, Turnstrasse 27, 73430 Aalen, Germany; siegfried.wahl@zeiss.com; 3 Institute for Ophthalmic Research, Eberhard-Karls University, Elfriede-Aulhorn-Strasse 7, 72076 Tuebingen, Germany; 4 Center for Ophthalmology, Eberhard-Karls University, Elfriede-Aulhorn-Strasse 7, 72076 Tuebingen, Germany; Focke.Ziemssen@med.uni-tuebingen.de

**Keywords:** microscope, ocular, 16S rRNA gene, sequencing, Illumina, eye, hygiene, microbiota

## Abstract

Microscopes are used in virtually every biological and medical laboratory. Previous cultivation-based studies have suggested that direct contact with microscope eyepieces increases the risk of eye infections. To obtain a deeper insight into the microbiota on oculars, we analysed 10 recently used university microscopes. Their left oculars were used for a cultivation-based approach, while the right oculars served for massive gene sequencing. After cleaning with isopropyl alcohol, the oculars were re-sampled and analysed again. All oculars were found to be contaminated with bacteria, with a maximum load of 1.7 × 10^3^ CFU cm^−2^. MALDI Biotyping revealed mainly *Cutibacterium* (68%), *Staphylococcus* (14%) and *Brevibacterium* (10%), with the most abundant species being *Cutibacterium acnes* (13%) and *Staphylococcus capitis* (6%). Cleaning reduced the microbial load by up to 2 log scales. Within 10 uncleaned and 5 cleaned samples, 1480 ASVs were assigned to 10 phyla and 262 genera. The dominant genera before cleaning were *Cutibacterium* (78%), *Paracoccus* (13%), *Pseudomonas* (2%) and *Acinetobacter* (1%). The bacteriota composition on the cleaned oculars was similar; however, it probably largely represented dead bacteria. In summary, used oculars were significantly contaminated with skin and environmental bacteria, including potential pathogens. Regular cleaning is highly recommended to prevent eye and skin infections.

## 1. Introduction

Surfaces regularly touched by humans become easily contaminated with microorganisms. Many recent studies have addressed the microbial load and associated health risks of frequently used objects, such as smartphones or money [1,2,3], transportation vehicles [4], restrooms [5] or hospital surfaces [6]. All of them were found to be colonized by a broad variety of bacteria of mainly human skin and epithelia origin, depending on how they are used and/or the respective human body parts they get in contact with. Transmission of pathogens is likely to occur and especially surfaces regularly touched with human hands must consequently be regarded as fomites [7]. Pathogenic and/or potentially pathogenic microorganisms may cause infections, particularly if there is close contact to the skin, mouth and eyes, and if devices are used by different persons.

To elucidate the bacterial load and hygienic relevance of optical devices, which are in physical proximity to the eyes, we recently performed an aerobic, cultivation-based study on used spectacles, which are remarkably widespread devices in the population [8]. We found significant amounts of bacteria, dominated by staphylococci, whereby many of the identified taxa represented potential pathogens that may cause skin and eye infections. Using a molecular approach, based on high-throughput 16S rRNA gene sequencing, we recently showed that the spectacle community is dominated by bacteria typical for the skin areas that are in physical contact with the spectacle frames [9]. These studies allowed a first insight into the bacteriota of personal ophthalmic objects in close contact to human skin and eyes. Even though the observed bacterial colonization may be problematic in clinical environments or for infection-susceptible people, the majority of the identified bacteria were assumed to be part of the normal, personal skin microbiota, and therefore unlikely to cause severe infections in healthy individuals.

However, sharing optical devices may be more problematic. Previous, cultivation-based studies [10] suggested that direct contact with microscope eye-pieces significantly increases the risk of reoccurring eye infections, such as conjunctivitis. 26% of the investigated oculars carried bacteria known to be pathogenic or potentially pathogenic, such as *Staphylococcus aureus* [10].

To come to a more comprehensive insight into the microbial community of shared ophthalmic objects, we examined the microbiota on used microscope oculars with a polyphasic approach, using gene sequencing and cultivation-based techniques. Our study represents the first comprehensive analysis of the microbial contamination on microscope oculars and we believe it provides a solid basis for a deeper understanding of the hygienic relevance of these optical devices, which are used in virtually every laboratory.

## 2. Materials and Methods

### 2.1. Cultivation-Based Analyses

The 10 light microscopes (Motic BA 310, and Leica DME, both Wetzlar, Germany) used for swab-sampling were taken from a security level 1 microbiology laboratory at Furtwangen University, Campus Villingen-Schwenningen. They are used for basic courses in practical microbiology, but not for specific research analyses. Sampling was performed in May 2019, immediately after a student laboratory course. These microscopes were selected because they were stored in the same room and were mainly used for the same purpose (teaching) and by similar users (students).

Preliminary analyses showed that separate sampling of lenses and plastic eyecups did not yield enough material for downstream analyses. Therefore, lenses and plastic eyecups of each single ocular were sampled with one swab, respectively.

**Figure 1:**
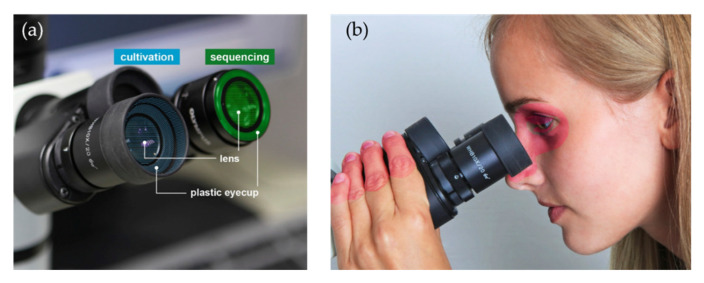
Sampled parts of the microscope oculars and skin areas that act as source for microbial contamination: (**a**) Each right ocular (lens and plastic eyecup) was sampled for sequencing-based analysis, each left ocular (lens and plastic eyecup) was sampled for cultivation-based analysis; (**b**) Skin and eye areas (highlighted in red) with probable contact to microscope oculars. Photographs with permission of Furtwangen University.

Each left ocular (lens and plastic eyecup) was sampled for the cultivation-based analysis (Figure 1a).

The sampled area was calculated by measuring the geometry of the ocular. Microbial loads were determined according to DIN 10113–1:1997–07—Part 1 [11]. Standardized sampling was performed in the university laboratory as described elsewhere [8], with a modified sample area to wetting medium ratio of 1.5:1 (1.5 mL medium was used per 1 cm^2^) in order to increase the cell concentrations.

Germ numbers were determined from that suspension by plating 50 μL, each on Tryptic Soy Agar (TSA; Carl Roth, Karlsruhe, Germany) as a non-selective medium for bacterial cultivation and Thioglycolate Agar (Merck KGaA, Darmstadt, Germany), which enhances the growth of non-stringent anaerobic/aerotolerant microbes, especially if applying prolonged cultivation times [12,13]. To detect fungi, 50 µL of suspension were plated on Malt Extract (Merck KGaA) and Sabouraud-4%-Glucose Agar (Carl Roth), respectively.

Aerobic cultivation conditions were as follows: 3 d for TSA Agar and 10 d for Thioglycolate Agar at 37 °C, respectively. 7 d at 30 °C for Malt Extract and Sabouraud-4%-Glucose Agar. Anaerobic cultivation was performed in an anaerobic jar using Anaerocult with indicators (Merck KGaA) for 7 d for TSA Agar and 10 d for Thioglycolate Agar at 37 °C, respectively. Germ numbers were determined after incubation, referred to the sampled area, and expressed as colony-forming units (CFU) cm^−2^. No anaerobic incubation was performed for the fungal growth media.

### 2.2. Identification of Microbial Isolates by MALDI Biotyping

From each agar plate showing microbial growth, a representative of each morphotype was subcultured and controlled for purity. A colony of each pure culture was suspended in 300 µL ultrapure water and stored at −80 °C until further processing. Samples were extracted and identified using a MALDI Biotyper system (MALDI Biotyper Microflex LT, Bruker Daltonics, Bremen, Germany) following the protocol for ethanol-formic acid extraction [14]. The volumes of formic acid and acetonitrile (both Carl Roth) were adapted as specified in the protocol for single, small colonies. The obtained protein spectral profiles were matched against the MALDI Biotyper reference database (software version 4.1.90, 8936 entries) and expressed as score values ranging from 0 to 3.0. According to the manufacturer, scores >1.7 indicate a reliable genus identification, scores >2.0 a reliable genus and probable species identification, and scores >2.3 a highly probable species identification. Detailed germ numbers and MALDI Biotyping results are provided in the Supplementary Table S1.

### 2.3. Sequencing-Based Analyses

Each right ocular (lens and plastic eyecup, Figure 1a) of the microscopes was sampled in a meandering pattern using dry, sterile Puritan Hydra Flock Swabs (Puritan Diagnostics LLC, Guilford, ME, USA). Swabs were broken off into RNA/DNA shield tubes (Zymo Research, Freiburg, Germany) with beads and stored at room temperature until further processing.

### 2.4. DNA Extraction

DNA was extracted and purified from the swab heads using the ZymoBIOMICS DNA Miniprep Kit (Zymo Research) following the manufacturer’s instructions with slight modifications. The samples within the shield were incubated at 50 °C for 20 min at 600 rpm, followed by five rounds of bead beating in a FastPrep 24 instrument (MP Biomedicals LCC, Santa Ana, CA, USA) for 1 min at 6.5 ms^−1^ and then placed on ice for 1 min.

After 2 min of incubation at room temperature, the DNA was eluted with 40 µL of 60 °C warm, DNA-free water. The flow-through was reloaded onto the same filter, and again incubated for 2 min. After centrifugation, an additional 10 µL of elution buffer were added onto the same filter, incubated for 1 min and centrifuged. The purified DNA was stored at −20 °C until further analysis.

### 2.5. Library Preparation

The V1–V3 region of the 16S rRNA gene was amplified using primers 63F (5′-CAGGCCTAACACATGCAAGTC -3′) [15] and 511R (5′-GCGGCTGCTGGCACRKAGT -3′) [16] (Eurofins Genomics GmbH, Ebersberg, Germany), with Illumina flow cell adapters (5′- TCGTCGGCAGCGTCAGATGTGTATAAGAGACAG -3′), yielding a PCR product of ~545 bp. We chose these primers to ensure data comparability with a previous study about the spectacle microbiota [9]. Moreover, these primers did not yield many unspecific PCR products. Most of the extracted samples were processed in duplicates. Triplicates were performed if the gel electrophoresis showed only weak bands. All samples were amplified on a Bio-Rad T 1000 Thermal Cycler (Bio-Rad Laboratories, Hercules, CA, USA) in a total reaction volume of 25 µL, containing 3 µL of template DNA, 15.05 µL of nuclease and DNA free water, 5 µL of 5 × KAPA High Fidelity Buffer (KAPA Biosystems, Wilmington, MA, USA), 0.6 µL of 10 mM KAPA dNTP Mix, 0.25 µL of 20 mg/mL BSA (Thermo Fisher Scientific, Darmstadt, Germany), 0.5 µL of KAPA High Fidelity Hot Start Polymerase, 0.3 µL of forward (10 µM) and 0.3 µL of reverse primer (10 µM).

The PCR profile was run as follows: 98 °C initial denaturation for 3 min, followed by 35 cycles of 98 °C for 30 s, 63 °C for 30 s, 72 °C for 60 s, and a final extension at 72 °C for 2 min. The DNA amplicons were verified by standard 0.8% agarose gel electrophoresis using Midori Green as DNA-dye (Biozym, Olderndorf, Germany). With each batch, water template control reactions were included. No PCR background contamination from either reagents and/or collection procedures was discovered. As positive controls, we used diluted (1:100) DNA from overnight cultures of *Escherichia coli* K12, extracted with the same DNA purification kit.

Clean-up of two or three pooled replicates of each PCR sample was performed using Agencourt AMPure XP Beads (BeckmanCoulter Inc., Krefeld, Germany) according to the Illumina library preparation protocol with changes in the bead to sample ratio of 0.7:1 [17].

For the following annealing step of the dual-index barcodes, we used the Nextera XT Index Kit v2 Set B and Nextera XT Index Kit v2 Set C adapters (Illumina Inc., San Diego, CA, USA) and followed the Illumina library preparation protocol with slight modifications. We used 5 µL of cleaned amplicon PCR product, with a unique combination of 4 µL index primer, each, and performed a 25 µL PCR reaction with eight cycles. Index PCR products were verified by standard 0.8% agarose gel electrophoresis and cleaned up as described above, with a bead to sample ratio of 0.8:1. The Bioanalyzer 2100 Instrument with the DNA High Sensitivity Kit (both Agilent Technologies Deutschland GmbH, Waldbronn, Germany) was used for the final PCR quality check. Subsequently, the DNA was quantified using a Qubit 2.0 Fluorometer (Thermo Fisher Scientific).

### 2.6. Sequencing

The library was adjusted to 3 nM (with 10 mM Tris buffer, pH 8.5), combined with 30% PhiX control (Illumina Inc.), and finally diluted to 4 pM. Sequencing was performed on an Illumina MiSeq platform using the MiSeq Reagent Kit v3 (600 cycle) (Illumina Inc.) with a quality score ≥30 and default settings. Sequence files were deposited at the European Nucleotide Archive (ENA) under the accession number PRJEB37105.

### 2.7. Cleaning Tests

To evaluate the efficacy in reducing the microbial load, oculars for both the cultivation-based and sequencing analyses were cleaned directly after sampling and re-sampled after 30 s residence time, as described above. The oculars were rubbed with sterile cotton swabs (Deltalab, Barcelona, Spain) wetted with 70% isopropyl alcohol, following the recommendations for microscope maintenance [18].

### 2.8. Bioinformatics

Sequences were processed with QIIME 2-2019.7 [19]. Raw sequence data were imported and demultiplexed using the cassava 1.8 paired-end and demultiplexed fastq format. The data were quality filtered, denoised and chimera-checked using the paired-end dada2 pipeline (–p-trunc-len-f 301 –p-trunc-len-r 257 trim-left-f 0 –p-trim-left-r 0) [20,21]. Referring to this pipeline, identified amplicon sequence variants are denoted as ASVs (Amplicon Sequence Variants). Taxonomic classification was performed with the feature-classifier plugin, trained with scikit-learn 0.19.1. [22] by the 63F/511R region using the SILVA 132 99% reference database [23]. This was followed by taxonomy-based filtering to remove mitochondrial and chloroplast sequences. Sequence alignment was created using mafft [24] with the phylogeny pipeline ‘align-to-tree-mafft-fasttree’. Following taxonomic classification, ASVs classified as mitochondria or chloroplasts were removed.

The EzTaxon database (16S-based ID, January 2020; https://www.ezbiocloud.net/) [25] was used for further identification of the relatively most abundant ASV sequences. Additionally, they were classified into risk groups according to the German Technical Rules for Biological Agents (TRBA) 466 [26].

Alpha- and beta-diversity analysis was carried out within QIIME 2 using an even sampling depth of 19250 sequences per sample.

For diversity metrics and generation of principal coordination analysis (PCoA) plots, we used the ‘diversity core-metrics-phylogenetic’. Alpha rarefaction curves, alpha-diversity metrics (‘observed’, ‘shannon’, ‘evenness’ and ‘faith’s phylogenetic diversity’) and beta-diversity (unweighted and weighted UniFrac distances) were analysed using the ‘alpha-rarefaction’, ‘alpha-group-significance’ and ‘beta-group-significance’ functions.

Significant associations between alpha-diversity metrics (within the metadata group ‘Cleaning’ were calculated within QIIME 2, using a non-parametric Kruskal-Wallis-Test with Benjamini-Hochberg multiple test correction. Pairwise comparison of beta diversity distances between the factor ‘Cleaning’ was performed employing permutational multivariate analysis of variance (PERMANOVA, 999 permutations).

All metadata, the unrarefied ASV table, and the taxonomic assignments are provided in Supplementary Table S2. 

### 2.9. Statistical Analyses 

Statistical analyses and graphical visualizations for the cultivation and sequencing analyses were performed in R 3.6.3 using the packages ‘phyloseq’ [27], ‘vegan (version 2.5-6)’ [28], ‘coin’ [29], ‘tidyverse’ [30] and ‘qiime2R’ [31]. Figures were created in R using ‘ggplot2’ [30] and ‘ggpubr’ [32]. For differences within the microbial counts, between anaerobic and aerobic cultivation and cleaned and uncleaned oculars, we used the Wilcoxon Signed Rank Test for differences between paired samples.

For sequencing, we processed 20 samples, whereby 15 samples (10 uncleaned and 5 cleaned) yielded sufficient sequences for downstream analyses. The 15 samples were rarefied using R to a level of 19250 sequences for even sampling depth (seed: 1121983).

## 3. Results

### 3.1. Cultivation-Based Results

To quantify and identify the cultivable, living microorganisms on microscope oculars, we performed a cultivation-based approach. While no fungi were detected, we found all investigated oculars to be significantly contaminated with bacteria.

**Figure 2:**
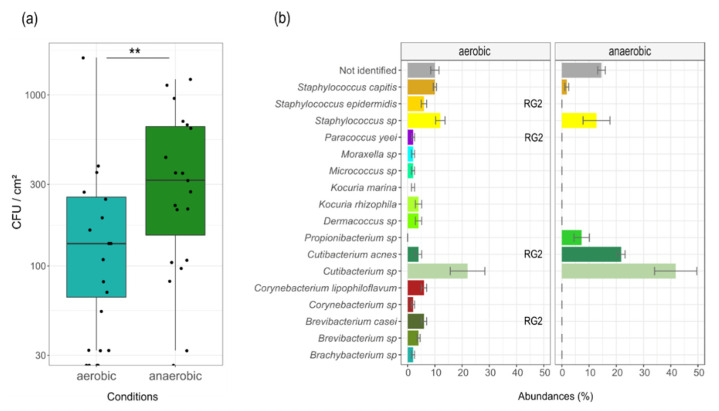
Microbial load and relative taxonomic abundances of bacteria isolated from ten uncleaned microscope oculars under two cultivation conditions: (**a**) Box-whisker plot showing the microbial counts (CFU cm^−2^) under two cultivation conditions and from two cultivation media (*n* = 10 oculars, each) before cleaning. Displayed are median, 25% and 75% quartiles, and outliers. Whiskers represent the lowest and highest microbial counts within the 1.5-fold of the interquartile range (IQR) (the 25% and 75% quartile). Asterisks mark a statistically significant difference between cleaned and uncleaned oculars (** *p* = 0.009), based on Wilcoxon Signed Rank Test; (**b**) Barplot of identified bacterial taxa isolated from the oculars before cleaning. Bars show the relative abundance for aerobic cultivation (n = 50 isolates) and anaerobic cultivation (*n* = 55 isolates). ‘Not identified’ indicates a MALDI identification score <1.7. ‘RG2’ indicates a risk group 2 classification according to German TRBA. Data are expressed as median ± standard deviation.

Averaged over all cultivation media showing bacterial growth, we determined a median bacterial count prior to cleaning of 235 ± 485 CFU cm^−2^ (median ± SD) for aerobic cultivation and 575 ± 727 CFU cm^−2^ for anaerobic cultivation. Cleaning reduced the bacterial load by ~2 log scales leaving 0 ± 9 CFU cm^−2^ for aerobic cultivation and 0 ± 230 CFU cm^−2^ for anaerobic cultivation. Differences between the cleaned and uncleaned oculars were significant (*p* = 3.05 × 10^−5^, aerobic cultivation; *p* = 3.82 × 10^−6^, anaerobic cultivation). Differences between the bacterial load of uncleaned oculars for the two cultivation conditions were also found to be significant (*p* = 0.009; Figure 2a). Bacterial contaminants on Malt Extract Agar were excluded from the evaluation. After cleaning, only 5 out of 10 oculars still showed microbial growth. The differences between aerobic and anaerobic cultivation were not significant (*p* = 0.125). 

MALDI-TOF fingerprints of 114 bacterial isolates (105 obtained before, 9 after cleaning) were used for identification at species or genus level. Ninety-two isolates (uncleaned) were reliably assigned on species or genus level (Figure 2b). All 9 isolates obtained after cleaning were reliably identified as cutibacteria.

In general, we found higher germ numbers but a lower number of genera under anaerobic conditions (11 genera for aerobic cultivation, 3 genera for anaerobic cultivation, Figure 2b).

The bacterial community was dominated by cutibacteria/propionibacteria among all anaerobically cultivated samples, before cleaning (71% on genus level; Figure 2b).

The next most common genera were staphylococci (aerobic: 28%, anaerobic: 15% on genus level) and brevibacteria (aerobic: 10% on genus level), followed by corynebacteria (aerobic, 8% on genus level). Further abundant taxa were *Kocuria* (aerobic: 6% on genus level) and *Dermacoccus* (aerobic: 4%). The remaining bacteria were all found with a frequency of 2%. Notably, four identified species are categorized as biosafety risk group 2 (Figure 2b).

### 3.2. Sequencing Results

Out of 1,983,441 raw sequences, we obtained 1,080,020 sequences after the dada2 pipeline. Five ‘cleaned’ samples did not yield enough sequences for downstream analyses and were excluded from further analyses. A total of 1,037,731 sequences were retained in the remaining 15 samples, with a mean of 72,912 (min. 19,250, max. 96,303) sequences per sample. After removal of singleton taxa and rarefication to 19,250 reads per sample (rngseed = 1,121,983) using R, we identified 1480 ASVs from 15 samples of uncleaned and cleaned microscope oculars (10 uncleaned, 5 cleaned). The taxonomic assignment of the ASVs revealed 10 bacterial phyla, 22 classes, 60 orders, 117 families and 262 genera.

### 3.3. Community Composition and Diversity

**Figure 3:**
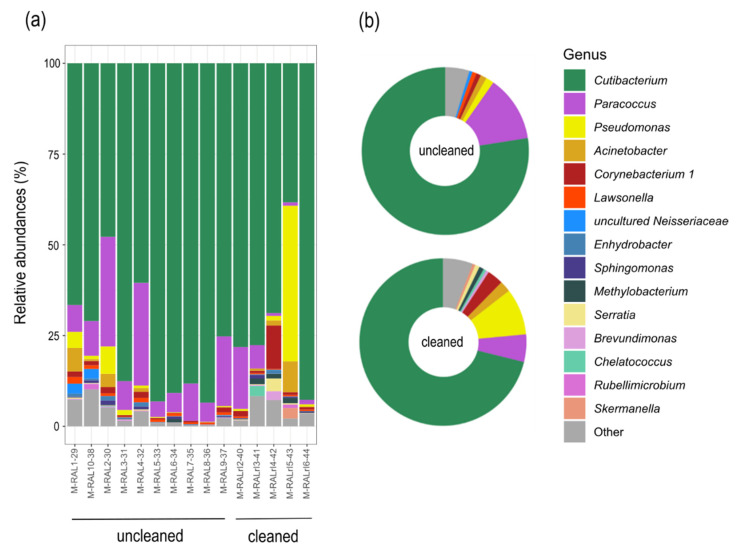
Stacked barplots of the relative abundances on genus level of uncleaned (*n* = 10) and cleaned (*n* = 5) microscope oculars: (**a**) Each bar represents one ocular sample; (**b**) Samples merged to pie charts for the factor ’uncleaned’ and ’cleaned’, representing the uncleaned and cleaned oculars. To facilitate comparison, only taxa with a relative abundance of >0.5% are displayed, the remaining taxa were summarized as ‘Other’.

According to the phylogenetic classification, most of the reads were affiliated with the genus *Cutibacterium* (78% uncleaned, 71% cleaned, Figure 3a). ExTaxon analysis revealed the most abundant sequences to be *Cutibacterium acnes* subsp. *defendens* (99% similarity). Other frequent genera were *Paracoccus* (13% uncleaned, 5% cleaned) and *Pseudomonas* (2% uncleaned, 9% cleaned), followed by *Acinetobacter* (1% uncleaned, 2% cleaned) and *Corynebacterium* (1% uncleaned, 3% cleaned). These top 5 genera comprised 91% (cleaned) to 94% (uncleaned) of all identified taxa. Figure 3 shows the relatively most abundant bacterial genera within the different samples. Less cutibacteria and more bacteria of the genus *Pseudomonas* were present on the cleaned oculars. However, this finding is strongly influenced by one of five samples, therefore it may not be representative.

In addition to *C. acnes*, ExTaxon analyses abundantly assigned sequences to *Paracoccus yeei* (100% similarity) and *Pseudomonas panacis* (100% similarity). The sequence of the most abundant ASVs within the genus *Acinetobacter* could not be classified down to species level, whereas *Corynebacterium* was affiliated with *Coynebacterium kroppenstedtii.*

When comparing the cultivation- and sequencing-based results, we found cutibacteria in similar ratios. However, we identified 21% staphylococci using the cultivation-based approach, but only 0.2% using molecular methods.

**Figure 4:**
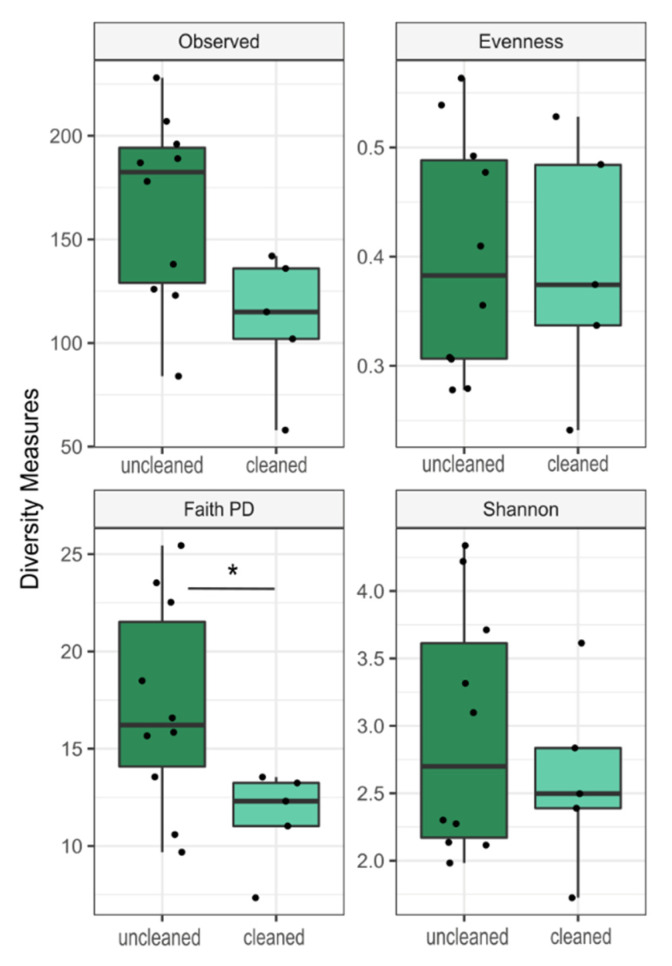
Comparison of alpha diversity measures between uncleaned and cleaned microscope oculars. Differences are shown by four indices (observed, Pielou’s evenness, Faith’s phylogenetic diversity and Shannon diversity). Points represent individual samples. Displayed are the median, the 25% and 75% quartiles and outliers. Whiskers represent the lowest and highest microbial counts within the 1.5-fold of the interquartile range (IQR) (the 25% and 75% quartile). Asterisks mark a statistically significant difference between cleaned and uncleaned oculars (* *p*-adjust = 0.04), based on Kruskal-Wallis Test with Benjamini-Hochberg multiple test correction.

Only faith’s phylogenetic diversity (faith pd) of the calculated Alpha-Diversity indices (Figure 4) showed a statistically significant difference in community composition between cleaned and uncleaned samples (Kruskal-Wallis, Benjamini-Hochberg corrected, *p* = 0.04). To assess beta-diversity, we calculated structural similarity and variation between the microbiota from cleaned and uncleaned microscopes using weighted and unweighted UniFrac-distances. No significant differences between cleaned and uncleaned oculars were detected (*p* > 0.05, PERMANOVA, 999 permutations).

## 4. Discussion

Frequently used objects and other regularly touched surfaces often carry a significant bacterial load and therefore represent fomites. This microbial contamination might lead to cross-contamination, if surfaces and devices are touched or used by different persons.

Our data provide evidence for significant microbial contaminations of microscope oculars as well, which are widely used optical devices in clinical or biological laboratories. Up to now, data on the microbial contamination of microscope oculars have been scarce, although there is a suggested relationship between their use and eye diseases [10]. Moreover, oculars are permanently exposed to the environment, so a diverse bacterial community was likely. As microscopes are touched regularly by hand, and the oculars are also likely to have direct skin contact, it was also safe to assume that typical dermal taxa would occur here (Figure 1b).

Our cultivation-based and molecular results were largely congruent. Indeed, we identified typical colonizers of human skin and mucous membranes as being dominant on the used oculars, such as staphylococci, corynebacteria, micrococci [33], and mainly cutibacteria [34]. In particular, the detected cutibacteria, which are slow-growing, aerotolerant anaerobes, are known to reside predominantly on facial skin and sebaceous glands [35], but are also found on the hands [36], and can therefore be easily transferred onto any touched surface [3,7].

Compared to other frequently touched surfaces, the detected numbers of cutibacteria were high [37,38]. This might be explained through the cultivation conditions that were used (aerobic and anaerobic, Thioglycolate Medium in addition to Tryptic Soy Agar, incubation time up to 10 d) [12], as well as the fact that the microscopes were sampled immediately after use.

Furthermore, some cutibacteria are known to develop biofilms, even on steel or silicone [39], which may lead to a better adherence to and/or persistence on surfaces, compared to other bacteria. 

As expected, the molecular approach allowed a more comprehensive insight, i.e., it unravelled a higher microbial diversity. Based on gene sequencing, the next most frequent genera alongside the cutibacteria were *Pseudomonas, Paracoccus* and *Acinetobacter.* ExTaxon analyses assigned the most frequent sequences to *Paracoccus yeei*, recently isolated from contact lenses and proposed to cause keratitis [40], and *Pseudomonas panacis*, an environmental species, recently isolated from rusty ginseng roots [41] and raw milk [42]. The sequence of the most abundant ASV within the genus *Acinetobacter* could not be classified at species level, whereas *Corynebacterium* was affiliated with *Coynebacterium kroppenstedtii*, a potentially opportunistic human pathogen [43].

In addition, cultures obtained from uncleaned oculars, and from both media, were identified as *Kocuria* and as brevibacteria, more specifically as *Brevibacterium casei*, which are typically associated with human skin [33,44,45]. Other bacteria that were found on the oculars represent ubiquitous or environmental taxa, such as *Paracoccus* [46] or *Brachybacterium* [47].

Many of the detected bacterial taxa are commonly found in the indoor and built environment [48]. They are associated with the human skin microbiome, comprising species also known to cause skin and eye infections [40,49,50]. Although we used media selective for fungi, no fungal growth was detected. This may be due to a shorter persistence of some fungi on surfaces, compared to bacteria [51].

Interestingly, using the cultivation-based approach we identified 21% staphylococci (on uncleaned oculars over all cultivation conditions), but only 0.2% using molecular methods. Staphylococci are known to thrive under a broad range of aerobic and anaerobic cultivation conditions [52], whereas, for instance, the optimal length of cultivation for *Cutibacterium acnes* is proposed to be around 7 to 10 days [13]. Therefore, staphylococci might have outcompeted other species during cultivation, leading to an overrepresentation in the cultivation results.

On the other hand, it is well known [53] that molecular methods can also discriminate certain groups of microorganisms, e.g., due to primer selectivity. In this study, we used primers targeting the V1–V3 region of the 16S rRNA gene that we had previously used for the analysis of the bacteriota on worn spectacles [9]. In that study, higher proportions of staphylococci were detected than here, demonstrating the potential of the used primers to amplify this group of bacteria. Clearly, future studies with other molecular methods and/or other primer combinations will be needed to corroborate or correct the results presented here and to help answer the question whether microscope surfaces select for certain microbial species. Nevertheless, our results strongly suggest that besides staphylococci, cutibacteria are an abundant bacterial genus on surfaces of microscope oculars. We recently reported a very similar trend for worn spectacles [9].

Notably, all isolated bacteria represented viable cells, i.e., they can potentially cause infections. To evaluate a probable pathogenic potential, the identified bacteria were categorized into biosafety risk groups. With *S. epidermidis*, *P. yeei*, *C. acnes*, and *B. casei* we found four potentially pathogenic bacterial species on the investigated oculars, i.e., species classified as risk group 2, which implies a probable infectious risk to humans.

*Propionibacterium* (*Cutibacterium*), *Staphylococcus* (especially *S. epidermidis)*, and Corynebacterium are part of the normal ocular microbiota and have previously been observed on eyelashes, eyelids and in tears [54,55]. Nevertheless, they are also known to be associated with blepharitis and bacterial keratitis [56,57]. We assume that bacteria are transferred easily from the skin, the area around the eyes, or the eyelashes to the oculars, and vice versa.

Importantly, many staphylococci comprise antibiotic resistant strains [58], such as MRSA (Methicillin-resistant *Staphylococcus aureus*) or MRSE (Methicillin-resistant *Staphylococcus epidermidis).* A study by Gerba and colleagues [38] showed that antibiotic resistant strains are typically present on frequently used and shared devices such as computer touchscreens. Other devices, such as the phones of health care workers, also carried nosocomial bacteria and antibiotic resistance strains [59]. Furthermore, frequently touched hospital and non-hospital surfaces were shown to carry a high proportion of multidrug resistant bacteria, mainly staphylococci [60]. Therefore, oculars should be considered as a potential reservoir for antibiotic resistant strains, too, which is of special importance in clinical environments, especially as it is known that many pathogens are persistent on surfaces for days or even months [51,61].

Cleaning with isopropanol had no notable effect on the taxonomic composition on the investigated oculars when considering the molecular data. Nonetheless, we found a lower Faiths phylogenetic diversity [62] on cleaned oculars, indicating more phylogenetic different taxa on uncleaned oculars, which matches the cultivation-based results. However, it is safe to assume that most of the detected sequences after cleaning stemmed from dead cells, because cultivation showed an ~2 log scale reduction of viable cells. These results strengthen the use of a biphasic analysis approach, combining cultivation-based and molecular methods. Future studies might also involve metagenomic approaches or the use of specific qPCR methods, which allow for a cultivation-independent detection of fungi, protozoa, such as acanthamoeba, or viruses. Detection of viruses, such as Herpes simplex or Varizella zoster, would be of particular interest, as many viruses cause severe eye infections [63,64,65]. A significant viral load on microscope oculars is likely, as studies showed that they can remain infectious on environmental surfaces for considerable time periods [51,66]. Even the (enveloped) new human coronavirus SARS-CoV-2 is detectable on plastic and steel surfaces for about 72 h [67].

Our findings corroborate and extend the findings by Olcerst [10] that microscopes carry potentially pathogenic bacteria and therefore may be associated with eye diseases of microscope users. After cleaning with 70% isopropyl alcohol and a 30 s residence time, only low numbers of cutibacteria were still detected on the oculars, which might be compensated by longer residence times. Clearly, cleaning reduced the microbial load significantly and therefore should be applied in a regular manner.

## 5. Conclusions

Microscope oculars carry a diverse bacterial load. Our study significantly extends previous findings about the bacterial load on microscope oculars by applying cultivation-based and cultivation-independent techniques. It provides a solid and comprehensive basis for a deeper understanding of the hygienic relevance of these widely used laboratory devices. We identified many viable taxa of human skin or mucosa origin, many of which are known to cause skin and eye infections. Due to the close skin and eyelash contact, microscope oculars must be regarded as fomites, especially when they are used by different individuals and in clinical environments. Cleaning with isopropyl alcohol reduced the microbial load significantly and should be performed on a regular basis. The dominant bacteria identified in our study appear as ideal test bacteria for antimicrobial efficacy testing of building materials and/or cleaning agents and strategies for microscope surfaces.

## Supplementary Materials

The following are available online at https://www.mdpi.com/2077-0383/9/5/1572/s1, Table S1: Rawdata Cultivation, Table S2: Rawdata Sequencing.
